# Visual Classical Conditioning in Wood Ants

**DOI:** 10.3791/58357

**Published:** 2018-10-05

**Authors:** A. Sofia D. Fernandes, C. L. Buckley, J. E. Niven

**Affiliations:** ^1^Department of Informatics, University of Sussex; ^2^School of Life Sciences, University of Sussex; ^3^Centre for Computational Neuroscience and Robotics, University of Sussex

**Keywords:** Behavior, Issue 140, Visual cues, appetitive conditioning, learning, memory, MaLER, *Formica rufa*

## Abstract

Several species of insects have become model systems for studying learning and memory formation. Although many studies focus on freely moving animals, studies implementing classical conditioning paradigms with harnessed insects have been important for investigating the exact cues that individuals learn and the neural mechanisms underlying learning and memory formation. Here we present a protocol for evoking visual associative learning in wood ants through classical conditioning. In this paradigm, ants are harnessed and presented with a visual cue (a blue cardboard), the conditional stimulus (CS), paired with an appetitive sugar reward, the unconditional stimulus (US). Ants perform a Maxilla-Labium Extension Reflex (MaLER), the unconditional response (UR), which can be used as a readout for learning. Training consists of 10 trials, separated by a 5-minute intertrial interval (ITI). Ants are also tested for memory retention 10 minutes or 1 hour after training. This protocol has the potential to allow researchers to analyze, in a precise and controlled manner, the details of visual memory formation and the neural basis of learning and memory formation in wood ants.

**Figure:**
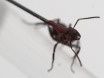


## Introduction

Insects have been extensively used as models for studying learning and memory formation^1^. A particularly successful vein of research involves using classical conditioning of restrained animals, which allows precise control over the cues being learned and permits researchers to investigate the neural mechanisms underpinning learning and memory. The majority of studies have focused on the appetitive classical conditioning of honeybee workers, *Apis mellifera*. The honeybee workers are trained to associate a CS with a US that evokes the UR. In this paradigm, originally developed by Takeda^2^ and Bitterman* et al.*^3^, the UR is the Proboscis Extension Reflex (PER), the US is sugar, and the CS is an odor. Bees learn the association between the CS and the US and can form a long-term memory of this association.

The original paradigm using the PER as the UR has been used to unravel the neural pathways underpinning olfactory learning in bees^4^. It has been modified in several ways to test learning of different sensory stimuli, including visual stimuli^5^,^6^,^7^, to incorporate aversive learning using suppression of the PER^8^ or the Sting Extension Reflex (SER)^9^ as the UR, and to test learning in other species, such as bumblebees^10^ and fruit flies^11^. Although memory formation with several modalities has been studied through classical conditioning, visual learning is still difficult to observe using this approach, even in species that show a high degree of visual learning ability during foraging, such as honeybees.

Recent studies have applied a similar approach to insects that do not have a proboscis, such as locusts, which perform the Palp Opening Response (POR)^12^, and ants, which perform the Maxilla-Labium Extension Reflex (MaLER)^13^. This has already revealed phase-specific learning abilities that match the phase-specific feeding strategies of the two different desert locust phenotypes, the gregarious and the solitarious forms, re-enforcing the idea that memory formation needs to match ecological needs^14^. Furthermore, studies on olfactory learning in ants has shown similarities between ants and honeybees in memory formation and retention, with long-term memory retention, 72 hours after training, being dependent on protein synthesis^15^.

These adaptations of the original paradigm have allowed learning and memory formation to be studied with many modalities and in several model species. This is essential for identifying mechanisms of memory formation common to the insects but also for identifying differences that reflect the particular ecology of learning and memory in different species. The main goal of the protocol we describe here is to provide a way to perform classical conditioning experiments using a visual conditional stimulus with a widely studied ant species, *Formica rufa*. It is issued from our study of visual learning in wood ants^16^, which is also an adaptation of visual classical conditioning paradigms.

## Protocol

### 1. Maintaining *Formica rufa* Colonies

Note: The wood ant (*Formica rufa*, L.) colonies used for this study were collected from Ashdown Forest, Sussex, UK (N 51 4.680, E 0 1.800). Within the UK, *F. rufa* colonies should be collected between July and August. A good proportion of the nest must be removed, including several hundred workers and brood, to ensure the colony is sustained and active for longer periods of time (up to one year). Permission should be sought from the concerned authorities before nest removal.

1. House the wood ant colonies in a wide box (around 135 cm x 70 cm x 35 cm) with walls covered with polytetrafluoroethylene (PTFE) resin (*e.g.*, Fluon) to prevent ants from escaping, at 21°C, under a 12-h light:12-h dark cycle.
  1. Provide sucrose (333 g/L) and water to the ants, using water dispensers. To ensure the ants’ diet is rich in protein, feed the colony with frozen crickets 2x a week. Spray the nest with water every day to keep it humid.
  2. To improve the sanitary conditions of the colony, add pine resin when necessary; pine resin reduces parasitism in wood ant colonies^17^. Place a small container (around 15 cm in diameter and 5 cm deep) inside the nest so that ants can deposit any dead colony members in it.
2. Keep ants under the afore-mentioned conditions for at least 2 weeks after collecting before attempting any experiments. Remove the sucrose from the nest to starve the ants 2 d prior to the experiments, thereby improving their motivation.

### 2. Selecting and Harnessing Ants

Note: For harnessing ants, a custom-made holder is needed. This can be constructed using modeling clay and a cardboard with a cut opening attached to it horizontally. The upper surface of the cardboard should be partially coated with wax, permitting insect pins to be attached during fixing. Careful handling is necessary for every step of this protocol (including maintenance, transport, and experiment), but in particular when harnessing ants, to avoid subjecting them to high levels of stress prior to training.

1. To select ants that are motivated to eat, place a slide with a drop of sucrose (200 g/L) inside a small box (around 14 cm x 8 cm x 5 cm) with walls covered with PTF resin to prevent escape. Do not select ants that are carrying wood or dead ants from the nest. Instead, take ants that are crawling up the walls of the nest, because these are more likely to be foragers trying to leave the nest to search for food.
2. Place each ant in the box and wait to see if it feeds on the sucrose drop. If it does, transfer it immediately to another empty box to prevent satiation.
3. Transfer each ant to a separate tube. Place each tube in the freezer for 1 - 2 min, or in crushed ice for up to 5 min, to immobilize the ant.
4. Place the immobilized ant in the holder, through the cut on the cardboard, by the joint between its head and thorax. Ensure the antennae remain on the side of the head, using insect pins embedded in the wax layer on top of the cardboard.
5. Use a custom-made heating element to wax the tip of an insect pin to the ant’s head, parallel to the cardboard. During this procedure, do not touch the antennae with the heating element wire to avoid incurring any damage to them.
6. After the wax dries, remove the insect pins holding the antennae and carefully remove the ant from the holder.
7. Fix the insect pin holding the ant in a modeling clay cylinder and fix a custom-made plastic holder beneath it, ensuring the ant maintains a typical standing posture and that the whole body is free to move except the head.
8. Keep the harnessed ants in darkness for at least 2 h prior to training.

### 3. Training and Testing

1. **Set-up, conditioned stimulus, and unconditioned stimulus**
  1. Conduct the experiments in a white acrylic box (50 x 50 x 50 cm), open at the front to permit access for the experimenter. Record the ant’s behavior with a camera.
    1. Place the camera with a macro lens directly above the ant, through a hole in the upper surface of the box.
    2. To reduce any extraneous visual cues, keep the room in darkness except for a light source pointing directly to the top of the acrylic box (two 26-W lights).
  2. Use as a visual cue (the CS) a bright blue (spectrum in **Figure S1**) cardboard rectangle (60 x 45 mm) attached at its center to a needle, connected to a syringe (2 mL) with which the US (sucrose 200 g/L) is manually delivered to the ant (Figure 1A and **1B**). Note: The sucrose solution can be made with any white sugar, provided that it does not have color and odor when dissolved in water.
  3. Conduct two types of training in parallel, paired and unpaired (see the explanation below), in a randomized order. Note: In this study, we did not pay attention to the nature of the visual cue. Only the association between the cue and the reward were taken into account. Color and shape were not tested, and it is likely that a CS with other features would produce similar results. Nevertheless, the blue color was picked because ants from the same genus have been shown to be sensitive to wavelengths that cover the blue color^18^.
2. **Paired training** Note: In paired training, the CS and US are presented in every trial, associated with each other (**Figure 1C**).
  1. Start recording the ants 10 s before presenting the CS to ensure that ants are not spontaneously performing MaLER before the presentation. If ants do perform MaLER during this period, postpone the trial for a few seconds. If any ant shows this behavior continuously, exclude it from the analysis.
  2. Move the syringe + CS in front of the ant for ~10 s, with the tip of the needle kept between the ant’s face and a maximum of 5 mm above and to each side. During this time, move the tip of the needle as close as possible to the ant’s head but without touching the antennae (Figure 1B). Note: Motion was included when presenting the CS because it has been shown to play a role in visual associative learning in honeybees^6^.
  3. Apply pressure to exude a single drop of sucrose from the syringe tip and place the drop of sucrose next to the antennae and mouthparts to allow the ant to detect the sucrose. Allow the ant to feed for about 5 s. Note: The amount of sucrose ants consumed in each trial was not controlled in these experiments. The drop of sucrose on which ants feed should be large enough to allow them to freely feed for about 5 s.
  4. Repeat this procedure 10x, with an ITI of 5 min. Note: In these experiments, ants were always turned to the right, with their right eye close to the open side of the acrylic box. Therefore, the CS always approached the ants from their right side. Though this does not invalidate learning, these experiments can be conducted by turning half of the ants to the left and half to the right, to avoid any possible lateralization effect.
3. **Unpaired training** Note: This training consists in presenting to the ants either the CS or the US separately, these two stimuli being, thus, dissociated from each other over time (Figure 1D).
  1. Start recording the ants 10 s before each trial.
  2. Present the CS in the same manner as in paired training but do not deliver the sucrose to the ant.
  3. After 2.5 min, deliver the US directly to the mouthparts (also touching the antennae) using a syringe without the CS attached.
  4. Start with a CS trial and repeat this procedure 10x for each type of trial, intercalating them with an ITI of 2.5 min.
4. **Testing** Note: A test should be conducted only 1x, either 10 min or 1 h after the last training trial; a test in which the ant is presented with the CS but not with the US could cause the extinction of the associative memory formed previously.
  1. Start recording the ants 10 s prior to testing.
  2. Present the CS to the ant for about 10 s.
  3. Ensure that the ant is still motivated to feed by delivering sucrose after the test.

### 4. Data Collection and Analysis

1. Record the ant’s behavior, from above, 10 s before each trial and during the CS and US presentations. Ensure that all trials and tests are recorded for posterior analysis.
2. Using the recordings made during training and testing, score the ants’ responses during the 10 s of CS presentation.
3. Separate the ants’ responses during the CS presentation into three types of behavior, depending on the extension and movement of the mouthparts: Full Extension with Movement (FEM) as if feeding, Full Extension without movement (FE) or Partial Extension (PE) of the maxilla-labium or maxillary palps (Figure 2A - **2D**). For analysis, group all MaLER types as a single response (Figure 2E).
4. Exclude any ant that did not feed in every trial and test. Note: For analysis, a statistical test that takes individual ants into account is recommended, thereby accounting for variability within individuals. When classifying ants’ responses during training in a binary manner (1 for positive responses and 0 for no response), logistic regression with mixed effects is advisable^16^,^19^. For comparing the proportion of ants responding in each training trial or test, a *G*-test or Fisher’s exact test is recommended, depending on the number of ants analysed^16^,^20^.

## Representative Results

During classical conditioning experiments, the CS must not induce a spontaneous response in the animals. In the experiments we conducted, only 3% - 4% of the ants performed MaLER in response to the visual cue on the first trial, prior to training. Ants that were undergoing a paired training performed increasingly more MaLER in response to the CS (Figure 3A; logistic regression, *N* = 51, degrees of freedom (df) = 507, z = 5.949, *p* < 0.01). The percentage of paired ants that respond to the CS plateaued around 50%, from the third trial onwards. On the contrary, unpaired ants showed no significant increase in MaLER during training (Figure 3B; logistic regression, *N* = 29, df = 287, z = 0.758, *p* = 0.45). The occurrence of MaLER in response to the visual cue was significantly higher during paired than unpaired training (logistic regression, *N* = 80, df = 796, z = -5.306, *p* < 0.01), which was true for every trial except the first (**Table 1**).

For examining their short- and a mid-term memory^15^, the ants were tested either 10 min or 1 h after the last training trial. For both tests, the proportion of ants performing MaLER in response to the CS was significantly higher when they had undergone paired rather than unpaired training (Figure 3C and **3D**; **Table 1**).

During training, individual ants showed substantial variation in the number and type of MaLER that they displayed (Figure 4A and **4B**). Only 14% of paired ants responded in every trial from the second or third trial onward, while most ants alternated between trials in which they responded and in which they did not. On those training trials in which ants responded, the degree to which they extended and moved their mouthparts varied. Therefore, we divided MaLER into three different types: FEM, FE, or PE. Typically, ants performed FEM or PE more often than FE. However, only a few ants performed consistently the same type of response; in most cases, ants showed little consistency on the type of MaLER they performed (Figure 4).

**Figure:**
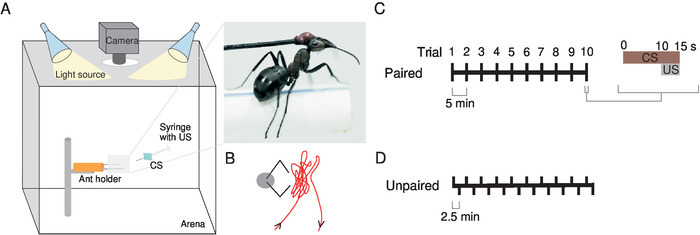

Figure 1**: Experimental set-up and training scheme.** (**A**) Fix the ant with wax to an insect pin attached to a modeling clay cylinder (orange) and place a custom-made holder underneath it to permit a naturalistic stance. Place it inside a white acrylic box illuminated by two light sources, directly underneath a camera. Use as the conditioned stimulus (CS) a cardboard (blue square) attached to the syringe that delivers the unconditioned stimulus (US), a sugar reward. The **inset** shows a close-up view of an ant in the holder. (**B**) The tip of the syringe needle is moved as close as possible to the ant's head but without touching the antennae as shown in this schematic. Train ants through either a (**C**) paired or (**D**) unpaired training. This figure has been modified from Fernandes* et al.*^16^. Please click here to view a larger version of this figure.

**Figure:**
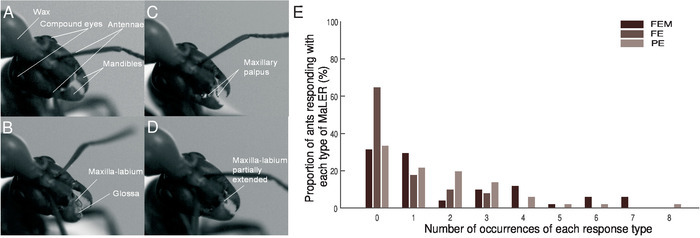

Figure 2**: The Maxilla-Labium Extension Response of wood ants.** Individual frames from video recordings show ants' mouth part movements during training. (**A**) This panel shows no response. (**B**) This panel shows a full extension (FE) of the maxilla-labium that terminates in the glossa. (**C**) This panel shows a partial extension (PE) with only the maxillary palpus visible. (**D**) This panel shows a partial extension (PE) of the maxilla-labium structures. (**E**) Ants undergoing paired training (*N* = 51) perform a full extension with movement (FEM; dark brown), an FE (mid-brown) or a PE (light brown). This figure has been modified from Fernandes* et al.*^16^. Please click here to view a larger version of this figure.

**Figure:**
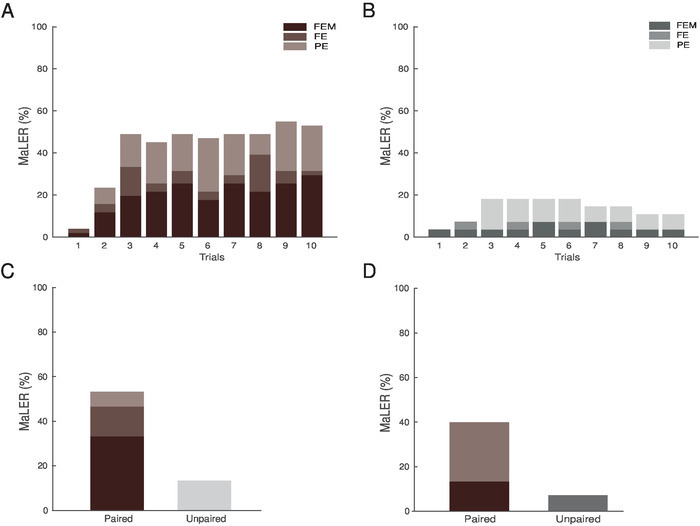

Figure 3**: Wood ants form associative memories of a visual cue paired with a sugar reward.** (**A**) The percentage of paired ants (*N* = 51) performing MaLER in response to the CS presentation significantly increased throughout training. (**B**) The percentage of ants performing MaLER did not increase significantly throughout unpaired training (*N* = 29). Ants were tested (**C**) 10 minutes (paired: *N* = 15; unpaired: *N* = 15) or (**D**) 1 hour (paired: *N* = 15; unpaired: *N* = 14) after the last training trial. The percentage of ants responding to the CS during and after paired or unpaired training are represented in brown or grey, respectively. The three types of MaLER are represented in dark (FEM), medium (FE), and light (PE) tones. This figure has been modified from Fernandes* et al.*^16^. Please click here to view a larger version of this figure.

**Figure:**
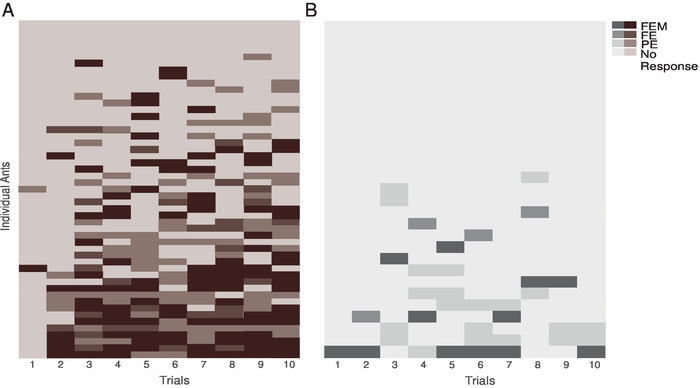

Figure 4**: Individual performance of ants during training.** These panels show the individual performances of (**A**) paired ants and (**B**) unpaired ants. The three types of MaLER are represented in dark (FEM), medium (FE), and light (PE) brown or grey. This figure has been modified from Fernandes* et al.*^16^. Please click here to view a larger version of this figure.

**Table:** 

**Trial**	**N**	**df**	**G (adjusted)**	**P**
1	80	na	na	>0.1
2	80	1	3.86	<0.05
3	80	1	8.41	<0.01
4	80	1	6.63	<0.01
5	80	1	8.41	<0.01
6	80	1	7.5	<0.01
7	80	1	10.69	<0.01
8	80	1	11.76	<0.01
9	80	1	17.13	<0.01
10	80	1	17.13	<0.01
10 min	59	1	5.5	<0.05
1 h	59	1	4.42	<0.05

**Table 1: Comparison of MaLER responses to the CS between ants that had undergone paired and unpaired training, for each trial and test.** The number of ants (N), degrees of freedom (df), *G*-test of independence (G) and *p*-value are shown. The first trial was analyzed with a Fisher's exact test. This table has been modified from Fernandes* et al.*^16^.


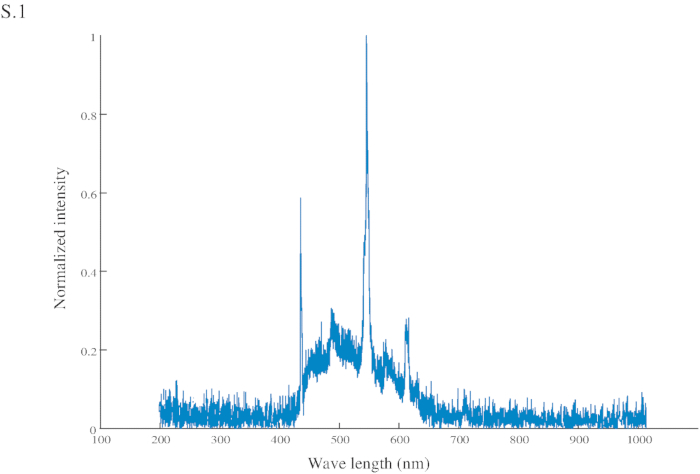
**Figure S1: Normalized intensity of the conditional stimuli (CS).** The CS has a peak intensity at 545 nm (in the green range) and another at 435 nm (in the blue range).

## Discussion

Classical conditioning is one of the most well-established paradigms to study learning and memory. The protocol we presented here is an adaptation of the paradigm designed for honey bee workers^2^,^3^ and subsequently used with several other species, such as bumblebees, fruit flies, which also use PER as a readout for learning^10^,^11^, and locusts and ants, which use POR and MaLER, respectively^12^,^13^. Using this protocol, it is possible to train harnessed wood ants to learn the association between a visual cue and a sugar reward and analyze the retention of this short- (10 min) and middle-term (1 hour) memory^16^.

In any behavioral experiment, it is necessary to take into account critical steps that can minimize the variability in the animals' responses. In the protocol presented here, several measures are taken to minimize variability before and during training. Prior to the start of experiments, the colony needs to be starved for at least two days and ants should be selected based on their willingness to eat from a sugar drop in the holding box. Selecting ants in this way is intended to maximize the chance of training ants that are motivated to feed. Careful handling is also an important consideration because it can help to reduce stress levels, which disrupts learning if it is too intense^21^. To this end, ants should be anesthetized with cold to stay motionless whilst being harnessed, because any movement (for escaping) during this procedure could be a source of stress. Furthermore, the contact between the ant and the wax should be minimal, avoiding contact between the antennae and the hot wax or wire, which could cause damage. Although these observations have not been analyzed formally, the antennae seemed to move with a specific pattern during learning.

During experiments, careful delivery of the sugar is also important to keep ants motivated. Again, whilst this has not been analyzed formally, abrupt food delivery seemed to cause additional stress to the ant, which in turn led to a lack of motivation and learning. Furthermore, the sucrose delivered during the training should be of a reduced concentration (200 g/L) to avoid satiation before the end of the training and testing. This allows MaLER to be a good candidate unconditional response because, together with a low spontaneous performance of this response to the visual cue, it also does not saturate over trials. Lastly, contrary to most classical conditioning studies^2^,^3^,^5^,^6^,^7^,^8^,^9^,^10^,^11^,^12^,^13^, we trained one ant at the time until the end of the experiment, leaving it in place between trials rather than removing it to test another ant. Training several ants together seemed to produce more variable results, which may be due to an increase in stress and/or conflict between visual information caused by the complete change of the scenery. To reduce the duration of each experiment, we used a 5-minute ITI instead of the 10-minute ITI used in most classical conditioning studies^16^. Although all these considerations should help ensure that the ants are motivated to feed and learn during training, some variability cannot be avoided. We recommend using ants that seem to have normal social, appetitive, and locomotion behavior and excluding ants from the analysis the moment they fail to feed on a training trial or a test.

The nature of the CS was not tested in this study. Although we have used a blue visual stimulus because ants of the same genus are sensitive to these wavelengths^18^, other colors might also be learned in association with a reward. Further experiments would be required to fully characterize the colors being seen and learned in this set-up. This is also true for different shapes and sizes of the visual cue. We have not tested if ants' spatial resolution would be sufficient for distinguishing the visual stimulus presented here at the distance from the ants' eyes it was presented at. Although wood ants' compound eyes have been described in terms of size and number of facets^22^, to our knowledge, their spatial resolution has not been fully described yet. However, this has been calculated for *Melophorus magoti*^23^. A similar characterization of the wood ants', or other tested insects' eyes would contribute to a clear investigation of the features of the visual cue being observed and learned by the animals. Furthermore, we included motion when presenting the visual stimulus to the ant because it has been shown to play a role in honeybee associative learning during classical conditioning^6^. However, this was also not tested in this study and, due to the different movement nature of flying insects compared to walking insects, differences between honeybee and ant visual classical conditioning could be observed.

On a final note, we were unable to examine long-term memory retention because ants did not survive being harnessed for such long periods after training. However, in subsequent sets of experiments, we have kept ants alive and motivated to eat and learn when harnessed and left them overnight in a dark and humid environment (placing a box over them). Therefore, this paradigm could be used to unravel long-term memory retention of wood ants, in addition to short- and middle-term memory.

With this simple procedure adapted from general classical conditioning paradigms, it is possible to study the acquisition and retention of visual memories in harnessed wood ants, which have been studied widely in paradigms using free-moving animals. This paradigm has the potential to be used for analyzing the neural basis of visual learning in a very well-established model for insect navigation.

## Disclosures

The authors have nothing to disclose.
